# Unsupervised consensus cluster analysis of [18F]-fluoroethyl-L-tyrosine positron emission tomography identified textural features for the diagnosis of pseudoprogression in high-grade glioma

**DOI:** 10.18632/oncotarget.14166

**Published:** 2016-12-24

**Authors:** Sied Kebir, Zain Khurshid, Florian C. Gaertner, Markus Essler, Elke Hattingen, Rolf Fimmers, Björn Scheffler, Ulrich Herrlinger, Ralph A. Bundschuh, Martin Glas

**Affiliations:** ^1^ Division of Clinical Neurooncology, Department of Neurology, University of Bonn Medical Center, Germany; ^2^ Stem Cell Pathologies Group, Institute of Reconstructive Neurobiology, University of Bonn Medical Center, Germany; ^3^ Department of Nuclear Medicine, University of Bonn Medical Center, Germany; ^4^ Neuroradiology, Department of Radiology, University of Bonn Medical Center, Germany; ^5^ Institute of Medical Biometry, Informatics and Epidemiology, University of Bonn Medical Center, Germany; ^6^ DKFZ –Division of Translational Oncology/Neurooncology, German Cancer Consortium (DKTK) & University Hospital Essen, Germany; ^7^ Clinical Cooperation Unit Neurooncology, MediClin Robert Janker Klinik, Bonn, Germany

**Keywords:** heterogeneity, FET-PET, pseudoprogression, glioblastoma, textural

## Abstract

**Rationale:**

Timely detection of pseudoprogression (PSP) is crucial for the management of patients with high-grade glioma (HGG) but remains difficult. Textural features of O-(2-[^18^F]fluoroethyl)-L-tyrosine positron emission tomography (FET-PET) mirror tumor uptake heterogeneity; some of them may be associated with tumor progression.

**Methods:**

Fourteen patients with HGG and suspected of PSP underwent FET-PET imaging. A set of 19 conventional and textural FET-PET features were evaluated and subjected to unsupervised consensus clustering. The final diagnosis of true progression vs. PSP was based on follow-up MRI using RANO criteria.

**Results:**

Three robust clusters have been identified based on 10 predominantly textural FET-PET features. None of the patients with PSP fell into cluster 2, which was associated with high values for textural FET-PET markers of uptake heterogeneity. Three out of 4 patients with PSP were assigned to cluster 3 that was largely associated with low values of textural FET-PET features. By comparison, tumor-to-normal brain ratio (TNRmax) at the optimal cutoff 2.1 was less predictive of PSP (negative predictive value 57% for detecting true progression, p=0.07 vs. 75% with cluster 3, p=0.04).

**Principal Conclusions:**

Clustering based on textural O-(2-[18F]fluoroethyl)-L-tyrosine PET features may provide valuable information in assessing the elusive phenomenon of pseudoprogression.

## INTRODUCTION

Despite state-of-the-art surgery, radiation therapy and chemotherapy, the prognosis of patients with high-grade glioma (HGG) is grim. In patients with the most aggressive and devastating form of HGG, glioblastoma [1], median overall survival is about 17 months. Considering the limited therapeutic options for patients with these tumors, it is important to detect tumor progression reliably, because otherwise, potentially efficacious therapies might be discontinued prematurely. In some cases, it is difficult to interpret post-therapeutic MRI alterations since true progression cannot be clearly distinguished from the so-called pseudoprogression, which may be due to tumor necrosis rather than due to tumor progression and therefore may reflect therapeutic efficacy [2]. So far, the diagnosis of pseudoprogression is built on increasing contrast-enhancement on MRI. This is similar for true tumor progression, but in the event of PSP contrast-enhancing lesions are stable or even regressive on subsequent MRI scans [3–8]. When increasing contrast-enhancing lesions on MRI indicate pseudoprogression, the current gold standard is to perform follow-up MRIs to evaluate for potential changes in lesion size over time. Consequently, the diagnosis of pseudoprogression is retrospective, requiring follow-up MRIs. Patient management would benefit from earliest diagnosis of pseudoprogression, ideally, when expanding contrast-enhancing lesions are detected for the first time. This is particularly important for patients with greatly increasing contrast-enhancing lesions and deteriorating clinical status. These patients might not be able to wait for 4-8 weeks for a follow-up MRI to decide whether secondary surgery or any other therapeutic adjustments are necessary.

Position emission tomography (PET) using radiolabeled amino acids such as O-(2-[^18^F]fluoroethyl)-L-tyrosine (FET) allows imaging of amino acid transport in brain tumors and has shown promise in distinguishing pseudoprogression from truly progressive tumor [9]. Some static and dynamic PET features have been shown to be strongly associated with early and late pseudoprogression [10]. As to static PET parameters, particularly the maximum tumor-to-normal brain ratio (TNRmax) at an optimal cutoff of 1.9 has been shown to be useful with a high sensitivity and specificity (sensitivity 84%, specificity 86%, negative predictive value 67%) in detecting true progression [11]. However, some patients with borderline TNRmax values remain subject to uncertainty when it comes to classifying to either true progression or pseudoprogression.

Besides the conventional markers derived from a PET image that reflect metabolism, which are usually variations of the standardized uptake value (SUV) within a region of interest (ROI), PET tracer uptake depends on several other physiological features pertinent to a tumor, such as perfusion, cell proliferation, tumor viability, hypoxia and aggressiveness [12]. Those properties account for tumor uptake heterogeneity. Textural PET markers are held to capture these properties by describing the tracer activity distribution within the tumor ROI [12]. Textural parameters assessed in ^18^F-FDG PET reflecting the texture of intratumoral tracer uptake (tumor uptake heterogeneity) have been shown to be prognostically relevant in several tumor etiologies, such as soft-tissue sarcoma, bone sarcoma, esophageal cancer and non-small cell lung cancer [12–14]. Additionally, it is suggested that textural markers might be valuable for tissue classification, particularly in separating malignant from benign tissue with a specificity as high as 99% [15].

Since HGG are heterogeneous tumors at histopathological and molecular levels [17], we investigated in a hypothesis-generating pilot study whether textural FET-PET features might be of value for drawing a distinction between true tumor progression and pseudoprogression. To address this, we retrospectively examined the predictive value of FET-PET parameters for detecting pseudoprogression in 14 patients with HGG using a set of textural parameters as compared with established PET features.

## RESULTS

### Patient characteristics

The study population comprised 14 patients (Table 1) with histologically proven HGG (GBM, n=11; WHO III, n=3). A methylated MGMT promoter was found in 12 and a non-methylated MGMT promoter in 2 patients. All patients underwent radiotherapy before PET investigation, either concomitant with chemotherapy or separated. Nine patients included in the study underwent FET-PET investigation during first-line treatment and 5 patients after relapse had occurred.

**Table 1:** Patient Characteristics

No	Cluster	Sex	Age at Dx (y)	Histologic Dx	MGMT methylated?	Line of therapy*	Treatment regimen until PET investigation	Concomitant dexamethasone treatment?	Wks from last Rx	Follow-up MRI + Clin.	Follow-up Time (m)	PFS (m)	OS (m)
**1**	1	m	29	AA	yes	1	P: B,RT+TMZ	no	7	stable	27.2	>26.7	>27.2
**3**	1	m	45	GBM*	yes	2	P: B,TMZ; 1R: R,RT,PC	yes	16	prog.	16.4	8.4	16.4
**4**	1	f	40	AOA	yes	4	P: pR; 1R: TMZ; 2R: TMZ; 3R: pR,RT,CCNU	no	34	prog.	126.4	>26.1	>126.4
**10**	1	m	43	GBM*	no	1	P: pR,RT,PC	no	37	prog.	24.1	8.0	24.1
**12**	1	m	70	GBM	yes	2	P: pR,RT+TMZ,TMZ; 1R: R,TMZ	no	139	prog.	45.1	4.1	45.1
**14**	1	f	68	GBM	yes	1	P: cR,RT+TMZ,TMZ	yes	10	prog.	23.4	>22.1	>23.4
**5**	2	f	49	GBM	no	1	P: pR,RT+TMZ,TMZ	no	52	prog.	34.1	4.6	34.1
**6**	2	m	61	GBM	yes	2	P: cR,RT+TMZ,TMZ; 1R: R,RT,CCNU/TMZ	no	25	prog.	23.5	>13.3	>23.5
**8**	2	m	60	GBM	yes	1	P: cR,RT+TMZ,TMZ	no	33	prog.	11.3	2.2	11.3
**13**	2	m	54	GBM	yes	1	P: pR,RT+TMZ	no	4	prog.	10.0	6.0	10.0
**2**	3	m	59	GBM	yes	1	P: cR,RT+TMZ/CCNU, TMZ/CCNU	no	95	stable	44.3	>21.7	>44.3
**7**	3	f	47	AA	yes	1	P: B,RT+TMZ,TMZ	no	25	stable	27.5	16.7	27.5
**9**	3	f	66	GBM	yes	1	P: cR,RT+TMZ,TMZ	no	48	prog.	21.7	5.1	21.7
**11**	3	m	50	GBM	yes	2	P: cR,RT+TMZ,TMZ; 1R: R,RT+CCNU/TMZ,CCNU/TMZ	no	41	stable	49.3	13.9	49.3

### Diagnosis of true tumor progression versus pseudoprogression

Four of 14 patients had confirmed PSP. Ten patients were regarded as having unequivocal progression (Table 1). All patients diagnosed with PSP had a methylated MGMT promoter whereas the MGMT promoter was methylated in 80% (8 of 10) in patients with true tumor progression. The mean time interval between initial MRI and follow-up MRI was 11 weeks.

### Identification of FET-PET-based subtypes

**Figure 1:**
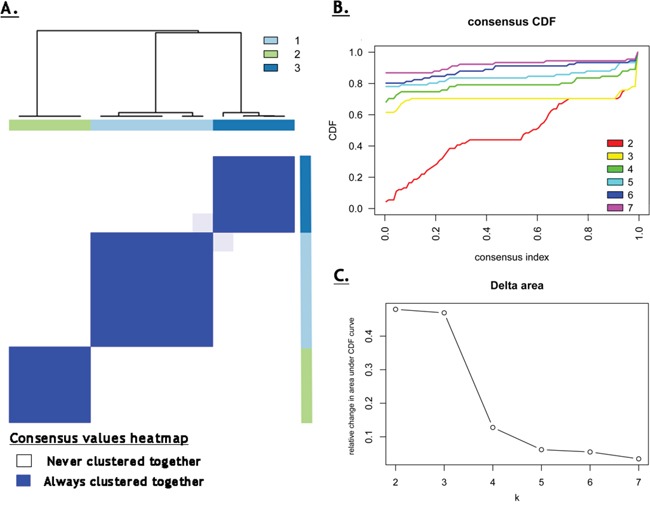
**A.** Consensus values heatmap, demonstrating a clearly delineated block structure for k=3, supporting a three-cluster solution; **B.** This is endorsed by the cumulative distribution function (CDF) curve, which approaches an ideal step function for k=3; **C.** The relative change in area under CDF curve illustrates that as k is increasing beyond k=3, there is a significant drop in the relative change in area under CDF curve, indicating an optimum at k=3.

Several methods served to detect the optimal number of clusters. For this purpose, we built a consensus matrix, which is obtained by measuring for each pair of patients, the proportion of clustering runs where 2 patients are clustered together based on the similarity of their FET-PET features. Descriptions of PET features used in this manuscript are given in Table 2. In the event of perfect consensus, the consensus matrix would be filled with 0 and 1 only. As shown in Figure 1a, the consensus matrix displays a well-defined 3-block structure for k=3, corresponding to 3 distinct cluster groups. To justify that k=3 corresponded to the optimal number of clusters, we compared the consensus matrix at k=2 through k=7 by using the CDF curve. The CDF curve plots the consensus distribution, which is a quantification of how entries of the consensus matrix are distributed within the range of 0 to1. Distributions containing only 0 and 1 would result in an ideal step function of the CDF curve. Here, we can see that for k=3 the ideal step function is approached (Figure 1b). The shape of the CDF curve hardly changes as k is increased past 3 (Figure 1b). The difference between 2 CDF curves (at k and k+1) is summarized by measuring the area under the CDF curves for k=2 through 7 and shown in Figure 1c. As k is increased, the area under the CDF curve stays approximately the same until k=3 and drops off significantly beyond that value. Any further increase in k does not come along with a corresponding marked increase in the CDF area, thus further supporting the choice of an optimal k=3. This result was confirmed by using the recently published PAC method, which was shown to be more accurate in determining the right number of k, where PAC was lowest at k=3, reflecting an optimal clustering with 3 groups. Of the 14 patients in our cluster cohort, 6 patients were assigned to cluster 1 (43%), and 4 patients each (29%) were assigned to cluster 2 and 3.

**Table 2:** Description of PET features

PET Feature	Explanation
Correlation	A measure of continuous areas of same or similar voxel values in an image. An image with high correlation values is usually associated with large areas of similar uptake intensities.
Coarseness	A measure of the intensity differences throughout the image.
COV	A normalized measure of dispersion of a frequency distribution (standard deviation divided by the mean value of the activity concentration in the tumor volume).
Contrast	A measure of local variations present in the image. A high contrast value indicates a high degree of local variation.
Complexity	Measures the uniformity of patterns versus rate of change in an image.
Entropy	Measures randomness of distribution, e.g. a homogenous matrix demonstrates low entropy.
Size Variation	Measures the difference of the grey value when going to the next voxel. It is high when the intensity changes very often between single voxels.
Intensity Variation	The intensity variation describes the variation of the intensity of different substructures.
Short Run Emphasis	Measure of consecutive pixels which have the same gray level intensity along a specific linear orientation. Fine textures tend to contain more short runs with similar gray level intensities.
Long Run Emphasis	Measure of consecutive pixels which have the same gray level intensity along a specific linear orientation. Coarse textures have more long runs with significantly different gray level intensities
Short Zone Emphasis	Measures the distribution of short zones as the difference of the grey value when going to the next voxel. It is high when the intensity changes very often between single voxels.
Long Zone Emphasis	Measures the distribution of long zones as the difference of the grey value when going to the next voxel.
Zone Percentage	Measures the percentage of zones of a given size.
SUV Mean	A measure of mean radiotracer accumulation in tumor lesions.
SUV Max	A measure of maximum radiotracer accumulation in tumor lesions.
TNR Mean	Mean tracer uptake in the tumor divided by that in normally appearing brain tissue.
TNR Max	Maximal tracer uptake in the tumor divided by that in normally appearing brain tissue.
TLU	The total lesion volume and its metabolic activity
Volume	The total lesion volume

### Assigning FET-PET features to each cluster

**Figure 2:**
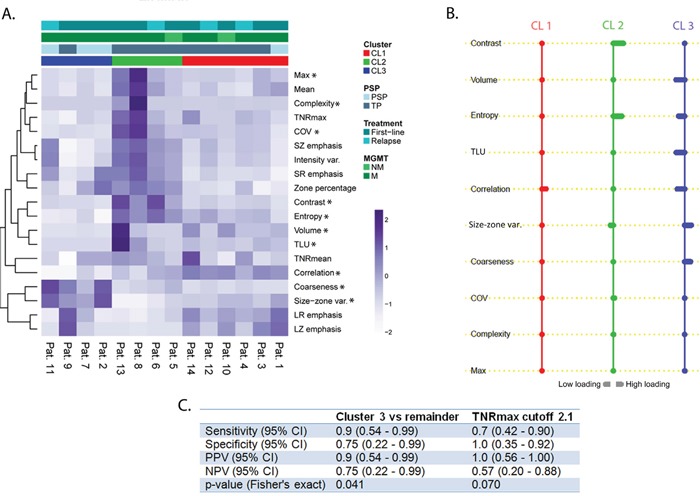
**A.** Heat map with patients ordered with regard to their cluster membership. On the vertical axis, the 19 FET-PET features are ordered by hierarchical clustering to demonstrate their association with each cluster; PET feature values are given as z-scores. Pat., patient; PSP, pseudoprogression; TP, true progression; MGMT, O-6-methylguanine-DNA methyltransferase; NM, not methylated; M, methylated; * indicates that a features belongs to the 10 most relevant features; **B.** Results of nearest shrunken centroid method indicating high loadings on textural features for cluster 2. The reverse association is observed in cluster 3. Cluster 1 was solely associated with high loading on Correlation. A rightward deflection signifies high loadings on that feature whereas a leftward deflection signifies the opposite. Cl1, cluster 1; CL2, cluster 2; CL3, cluster 3; **C.** Performance of cluster 3 vs. TNRmax in detecting true progression. CI, confidence interval; PPV, positive predictive value; NPV, negative predictive value

To identify FET-PET features associated with each cluster, we used the nearest shrunken centroid method called PAM. Predictor discovery by PAM identified 10 PET features out of 19 with at least one nonzero component. This implies that those selected features simultaneously distinguish all clusters from each other. Figure 2a shows a heat map of all hierarchically clustered features corresponding to each cluster and Figure 2b shows the shrunken differences for the 10 PET characteristics differentially regulated across the 3 clusters. Of those, 8 characteristics are textural features (Contrast, Entropy, Correlation, Size-zone var., Coarseness, Volume, COV, and Complexity) and 2 are recognized as conventional (TLU, Max). Notably, the upper 7 (Figure 2b) of those 10 features provide the most distinct separation among clusters: Contrast, Volume, Entropy, TLU, Correlation, Size-zone var., and Coarseness. From the distribution of FET-PET features across clusters using PAM, it becomes evident that cluster 2 was particularly associated with high values of the textural characteristics Contrast and Entropy (Figure 2b). As increased values of both features have been tied to intratumoral tracer uptake heterogeneity [12], the cluster 2 phenotype was designated “high heterogeneity cluster”. Cluster 3 was largely associated with inverse loadings of FET-PET textural features as compared with cluster 2 - except for the feature Coarseness -, most strikingly Entropy, Correlation, and Size-zone var. With high intratumoral tracer uptake heterogeneity, Entropy and Correlation are known to be increased whereas Size-zone var. decreased [12]. Cluster 3 was thus named “low heterogeneity cluster”. Interestingly, TLU was also comparably downregulated in this cluster. As opposed to cluster 2 and 3, cluster 1 had the least variability in features. Only the feature Correlation was considerably upregulated. As such, cluster 1 was defined as “intermediate cluster”.

### Pseudoprogression and cluster assignment

All of the patients assigned to cluster 2 (4 out of 4) and 5 out of 6 of cluster 1 were diagnosed with progression. Contrarily, 3 out of 4 patients with pseudoprogression fell into cluster 3 (Figure 2a). TNRmax differed significantly (p=0.039) between patients diagnosed as pseudoprogression (mean, 1.9; range, 1.7-2.1) and true progression (mean, 2.6; range, 1.2-4.4).

**Figure 3:**
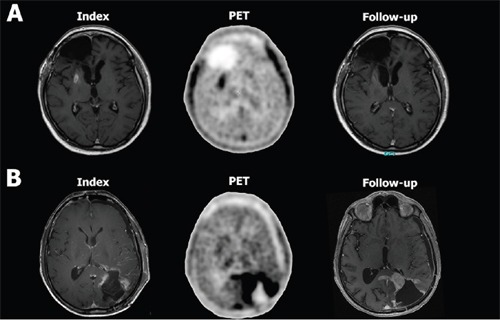
PET image with the corresponding MRI scans from a patient with pseudoprogression (patient 2, A) and true progression (patient 13, B) Figure 1A shows exemplary the index T1 contrast-enhanced (CE) MRI scan where a new CE lesion in the right-sided basal ganglia appeared for the first time. The PET scan next to it shows an increased FET uptake in that area. On follow-up, the CE lesion regressed spontaneously, indicating pseudoprogression. Figure 1B shows exemplary the index T1 CE MRI scan with a new CE lesion around the resection cavity in the posterior lobe. The corresponding PET scan shows extensive FET accumulation far beyond the CE lesion. In the follow-up MRI scan, the index CE lesion increased considerably and thus confirmed true progression. By a mere visual comparison of the PET images from the patient with pseudoprogression **A.** with that from the patient with true progression **B.** one can not infer that there is any difference in tracer uptake. Assessing the texture of tracer uptake, however, provides more information than can be delineated by visual inspection.

In Figure 2c, the ability of performance metrics (such as sensitivity, specificity, etc.) in diagnosing true progression is compared between a clustering based classifier (cluster 3) and the conventional classifier (TNRmax). The cluster 3 classifier seems to be stronger associated with the detection of true progression (p=0.041) compared to TNRmax (p=0.07). Cluster 3 provided a high sensitivity and specificity (90% and 75%, respectively) for detecting true progression with a negative predictive value (NPV) of 75%. Similarly, TNRmax provided high values for specificity and sensitivity (70% and 100%, respectively), yet, at the cost of a low NPV (57%). Exemplary PET images with the corresponding MRI scans for pseudoprogression and true progression are given in Figure 3.

### Putative prognostic value of clusters

**Figure 4:**
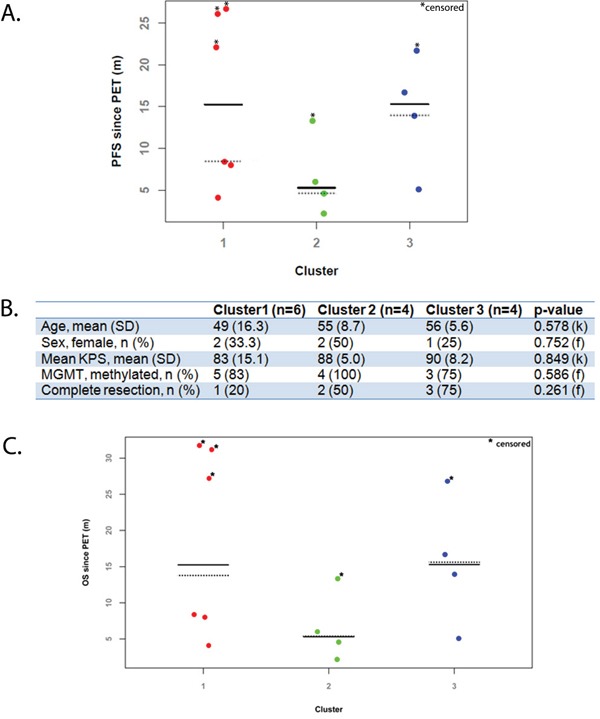
**A.** Dot plot of progression-free survival (PFS) by cluster groups; **B.** Distribution of prognostic factors by cluster. C. Dot plot of overall survival (OS) by cluster groups; k, Kruskal-Wallis test; f, Fisher's exact test; MGMT, O-6-methylguanine-DNA methyltransferase; KPS, Karnofsky performance status, SD, standard deviation; n, number; Solid line indicates median PFS values; Dotted line indicates median PFS values measured by Kaplan-Meier method.

As shown in Figure 4a, cluster 2 patients have a lower median PFS (solid line) when compared with the remaining clusters (5.3 months vs. 14.6 months in cluster 1 and 15.3 months in cluster 3). When calculating median PFS using the Kaplan-Meier method – accounting for censored values – cluster 2 (dotted line) remains the one with the lowest median PFS (4.6 months vs. 8.4 months in cluster 1 and 13.9 months in cluster 3). Figure 4c shows that a similar finding was observed with the overall survival data since PET. Figure 4b illustrates that this effect cannot be explained by differentially distributed prognostic factors among clusters, given a balanced distribution of prognostic factors.

## DISCUSSION

The results of this pilot study suggest that HGG patients with suspected pseudoprogression may be classified into 3 distinct clusters, solely based on a set of textural FET-PET features. Most of the patients assigned to cluster 3 had pseudoprogression while all patients assigned to cluster 2 had true tumor progression. Thus, textural FET-PET feature analysis might lend itself as a novel useful non-invasive tool, besides the frequently used TNRmax to distinguish pseudoprogression from true tumor progression in patients with HGG.

When we compared the value of pseudoprogression prediction using a cluster-based classifier (cluster 3), that was based on textural PET features, against the most widely used PET marker TNRmax [9, 11], only the cluster-based classifier was significantly associated with pseudoprogression detection. In addition, compared to TNRmax, NPV was higher with the cluster-based classifier, cluster 3. However, the significance of this analysis is limited given the retrospective and explorative nature of this study and its very limited sample size. Nevertheless, this approach is novel, the results are promising, and encourage to analyze the diagnostic value of textural markers in a larger cohort of patients.

Out of a set of 19 FET-PET features encompassing conventional (among others TLU, TNRmax, and TNRmean) as well as textural features, only 10 features separated all 3 clusters from one another. Of those 10, 7 features, namely Contrast, Volume, Entropy, TLU, Correlation, Size-zone var., and Coarseness were most differentially regulated among clusters and all of the latter 7 – except for TLU and Volume - are considered textural PET markers [12]. These textural features reflect intratumoral uptake heterogeneity and may be used to quantify tumor heterogeneity [12]. The degree of intratumoral heterogeneity is suspected to be a prognostic factor [18]. Some textural markers such as Entropy and COV have been shown to be prognostically relevant in systemic tumors [12, 19]. Intriguingly, cluster 2, which included only patients with true progression, exhibited high values of heterogeneity markers (particularly Contrast and Entropy). By contrast, cluster 3, which included largely patients with confirmed pseudoprogression, was associated with low values of heterogeneity markers. On the other hand, TLU, the only non-textural marker of the 7 highly differentially regulated FET-PET-features, has been shown to be negatively correlated with prognosis and - compared to other conventional PET features - a stronger predictor of outcome in systemic tumors [20, 21]. Interestingly, TLU was inversely associated with cluster 3, supporting that the cluster assignment based on our set of PET features might carry prognostic implications.

Similarly, in a recently published retrospective study [22] of patients with HGG, who received FET-PET prior to first-line treatment, 3 of the textural markers assessed here, namely Complexity, Contrast and Coarseness, were shown to be possibly correlated with survival. In our very small-sized patient cohort, cluster 2 patients showed the lowest median PFS and OS compared to patients from the other clusters. Notably, canonical prognostic markers were similarly distributed among clusters and are not suited to explain this observation. However, survival times varied considerably among patients sharing the same cluster and the sample size was too small to draw strong conclusions from this pilot data. In addition, it should be mentioned that our cohort consisted of 5 patients who underwent PET after relapse had occurred. With the other patients included in the first-line therapy, our cohort was heterogeneous to some degree although those patients included after relapse were treated again with alkylating (radio) chemotherapy. This cohort heterogeneity and the issue that treatment at recurrence might further account for varying PET data makes interpretation difficult. Nevertheless, because our findings might indicate a putatively prognostic value of clusters defined by textural FET-PET markers reflecting intratumoral uptake heterogeneity, a prospective study with a larger patient cohort validating our results is warranted.

In summary, this work provides a novel and interesting approach to FET-PET based identification of pseudoprogression, however, as mentioned above, by virtue of the small sample size interpretation of our results is limited and calls for validation in larger and systemic analyses.

## MATERIALS AND METHODS

### Patients

For this retrospective analysis, the patient files of the Division of Clinical Neurooncology were searched for pathohistologically confirmed HGG patients meeting the following characteristics: (1) patients experiencing increasing contrast-enhancing lesions on MRI (+25% in 2 perpendicular diameters) and/or any new lesion according to RANO [23] (minimum lesion size >10 mm) more than 4 weeks after the end of radiotherapy, (2) patients having a routine FET-PET following detection of increasing contrast-enhancing lesions, (3) after initial MRI and FET-PET, a further contrast-enhanced MRI ensued at least 4 weeks later without change of therapy. O-6-methylguanine-DNA methyltransferase (MGMT) promoter methylation status was tested using pyrosequencing [24]. This study was approved by the institutional ethics committee of the University of Bonn Medical Center.

### PET imaging with 18F-FET

Data were acquired with a Biograph Sensation 2 PET/computer tomography (PET/CT) scanner (Siemens Medical Solutions). The axial and transverse fields of view were 16.2 and 58.5cm respectively. The transverse resolution of the scanner was about 6.5mm, whereas the axial resolution was 6.0mm, both at a radius of 10mm. The computer tomography (CT) component was a 2-slice spiral CT scanner. About 20 minutes after the intravenous injection of approximately 200 MBq of FET, the patient was placed in the scanner. Low-dose CT of the head (caudocranial) was performed followed by the PET scan of the same area in a single bed position with 20 minutes acquisition time. The CT data were reconstructed in 512 × 512 pixel matrices. PET data were reconstructed into 256 × 256 matrices using the iterative attenuation-weighted ordered subset algorithm implemented by the manufacturer using 4 iterations and 16 subsets. Attenuation and scatter correction was performed using the CT data. Final voxel size was 5.3mm x 5.3mm x 5mm. All patients gave written and informed consent to the imaging procedure.

### PET data analysis

Image data were transferred to an Interview Fusion Workstation (Mediso Medical Imaging System, Budapest, Hungary). Firstly, co-registration between PET and CT images was performed. Tumor volume was manually delineated on PET images. For background assessment, 5 regions of interest (ROIs) with a fixed diameter of 15mm were placed on normally appearing cortex area, 2 on the frontal lobe, 2 on the occipital lobe and 1 on the contralateral region to the tumor. A mean value was then calculated for these ROIs. In addition, a semiautomatic segmentation in PET was performed based on background activity; to this end, the tumor delineation cutoff was set as 1.6 times the mean value of background ROIs. For the assessment of tumor uptake heterogeneity, 13 textural heterogeneity PET parameters were estimated, namely, Coefficient of Variation (COV) [19], Entropy, Correlation, Contrast, Size-zone variability (Size-zone var.), Intensity variability (Intensity var.), Coarseness, Complexity [25], Short Zone Emphasis (SZ emphasis), Long Zone Emphasis (LZ emphasis), Zone Percentage, Short Run Emphasis (SR emphasis), and Long Run Emphasis (LR emphasis) [12, 26]. All parameters were assessed in 3-dimensional volumes. In addition, the following 6 conventional PET parameters were evaluated: mean SUV (Mean), maximum SUV (Max), Morphologic Volume of the Lesion (Volume), Total Lesion Uptake (TLU) as product of lesion volume and mean uptake in the lesion analog to the total lesion glycolysis in glucose PET, mean tumor to background ratios (TNRmean) and maximum tumor to background ratios (TNRmax).

### Diagnosis of true progression

The diagnosis of tumor progression was made when progressive contrast-enhancing lesions according to RANO criteria [23] were noted on initial MRI and when further progression of contrast-enhancement ensued on a follow-up MRI at least 4 weeks later. By contrast, the diagnosis of pseudoprogression was applied when the follow-up MRI showed stabilization or regression of the contrast-enhancing lesions, provided that neither clinical worsening nor change in treatment ensued in the interim. In all patients, MRI scan analysis was carried out by an experienced neuroradiologist and another independent investigator.

In both the event of true progression and pseudoprogression, progression-free survival (PFS) was defined as the time between PET investigation and next progression as defined per RANO after the follow-up MRI used to confirm either true progression or pseudoprogression.

### Subtype discovery

Unsupervised consensus clustering was used for class discovery to uncover groups of items sharing FET-PET characteristics. Consensus clustering is a class discovery technique for the detection of unknown possible clusters consisting of items with similar intrinsic features [27]. Being distinct from conventional clustering methods, it provides quantitative evidence to determine the number and membership of clusters. To apply this method on our dataset, we first standardized FET-PET features to obtain z-scores. This was followed by subsampling 80% of items and PET features 10 000 times and partitioning each subsample up into k=7 groups (k represents the number of clusters) by the agglomerative hierarchical clustering algorithm using Pearson correlation distance. For each k, a consensus matrix was filled with consensus values, defined as the proportion of clustering repetitions in which 2 items are classified together. To determine the optimal number of k, we drew upon empirical cumulative distribution function (CDF) plots to find the k at which the distribution reached an approximate maximum, indicating optimal stability. For illustration purposes, it may be apt to assume that we observe the outcome of this kind of clustering for only 2 patients. Based on their PET features, 2 hypothetical patients are given a certain consensus index that may lie between 0 and 1. The CDF is an accumulation of all observed consensus indices for all patients at a given k. Consequently, the CDF is a measure of how well clustering turned out to be at a given k.

To validate the so obtained optimal number of clusters, we applied the proportion of ambiguous clustering (PAC) method [28].

To identify a minimal subset of PET features that succinctly characterizes each cluster, we used the nearest shrunken centroids method called predictive analysis of microarrays (PAM) [29]. For this purpose, we used 10-fold cross-validation to determine the amount of shrinkage at which the error rate was minimized.

### Statistical analysis

To assess cluster stability in the unsupervised analysis, along with performing consensus clustering over 10 000 iterations we used the CDF and CDF progression graphs to detect the optimal number of clusters. Furthermore, we relied on PAC to confirm our choice. To compare clinical and molecular data across clusters, we used the Kruskal-Wallis test for continuous variables and the Fisher's exact test for categorical variables. Moreover, logistic regression and Fisher's exact test for 2 × 2 contingency tables were performed to assess the association of pseudoprogression with cluster assignments. A p-value below 5% was considered significant. Statistical analysis was carried out using Stata (release 14.0; StataCorp LP) and R statistical software (version 3.2.4).
